# eNOS Protects from Atherosclerosis Despite Relevant Superoxide Production by the Enzyme in apoE^−/−^ Mice

**DOI:** 10.1371/journal.pone.0030193

**Published:** 2012-01-23

**Authors:** Padmapriya Ponnuswamy, Angelika Schröttle, Eva Ostermeier, Sabine Grüner, Paul L. Huang, Georg Ertl, Ulrich Hoffmann, Bernhard Nieswandt, Peter J. Kuhlencordt

**Affiliations:** 1 Medizinische Klinik I/Herz-Kreislaufzentrum, University of Würzburg, Würzburg, Germany; 2 Rudolf Virchow Zentrum für Experimentelle Biomedizin, DFG-Research Center for Experimental Biomedicine, University of Würzburg, Würzburg, Germany; 3 Division of Cardiology, Massachusetts General Hospital and Harvard Medical School, Boston, Massachusetts, United States of America; 4 Division of Vascular Medicine, Medizinische Poliklinik, Standort Innenstadt, Ludwig Maximilians University, Munich, Germany; Institut National de la Santé et de la Recherche Médicale, France

## Abstract

**Background:**

All three nitric oxide synthase (NOS) isoforms are expressed in atherosclerotic plaques. NOS enzymes in general catalyse NO production. However, under conditions of substrate and cofactor deficiency, the enzyme directly catalyse superoxide formation. Considering this alternative chemistry, the effects of NOS on key events in spontaneous hyperlipidemia driven atherosclerosis have not been investigated yet. Here, we evaluate how endothelial nitric oxide synthase (eNOS) modulates leukocyte/endothelial- (L/E) and platelet/endothelial- (P/E) interactions in atherosclerosis and the production of nitric oxide (NO) and superoxide by the enzyme.

**Principal Findings:**

Intravital microscopy (IVM) of carotid arteries revealed significantly increased L/E-interactions in apolipoproteinE/eNOS double knockout mice (apoE^−/−^/eNOS^−/−^), while P/E-interactions did not differ, compared to apoE^−/−^. eNOS deficiency increased macrophage infiltration in carotid arteries and vascular cell adhesion molecule-1 (VCAM-1) expression, both in endothelial and smooth muscle cells. Despite the expression of other NOS isoforms (inducible NOS, iNOS and neuronal NOS, nNOS) in plaques, Electron Spin Resonance (ESR) measurements of NO showed significant contribution of eNOS to total circulating and vascular wall NO production. Pharmacological inhibition and genetic deletion of eNOS reduced vascular superoxide production, indicating uncoupling of the enzyme in apoE^−/−^ vessels.

**Conclusion:**

Overt plaque formation, increased vascular inflammation and L/E- interactions are associated with significant reduction of superoxide production in apoE^−/−^/eNOS^−/−^ vessels. Therefore, lack of eNOS does not cause an automatic increase in oxidative stress. Uncoupling of eNOS occurs in apoE^−/−^ atherosclerosis but does not negate the enzyme's strong protective effects.

## Introduction

Atherosclerosis is a consequence of chronic inflammation of the vessel wall [1]. One of the key events is the recruitment of leukocytes and the adhesion of platelets to the endothelium overlying the plaque [2], [3]. Nitric oxide (NO), an important physiological regulator of vascular homeostasis is implicated in the pathophysiology of atherosclerosis [4]. In atherosclerotic plaques all three NO-synthase (NOS) isoforms are expressed: the neuronal (nNOS, NOSI), the inducible (iNOS, NOSII) and the endothelial (eNOS, NOSIII)[5]. In contrast, in normal arteries eNOS is the predominant NOS isoform [6]. While all NOS isoforms produce NO by catalytic conversion of L-arginine to NO and L-citrulline, the isoforms differ in catalytic activity, gene regulation and the cellular compartment. Complicating the interpretation of the role of NOS, all three isoforms can themselves produce superoxide (termed “uncoupling”), instead of NO, when substrate or cofactor is limited [7], [8], [9]. eNOS and nNOS are constitutive enzymes producing low concentrations of NO. We previously showed that both, eNOS and nNOS significantly inhibit atherosclerosis in apoE^−/−^ mice [10], [11]. In contrast to these protective effects, iNOS produces high concentrations of NO, which serves important roles in host defence against tumor cells and pathogens, but also contributes to tissue damage in chronic inflammation and increases apoE^−/−^ atherosclerosis [12], [13].

Pharmacological inhibition [14] and genetic deletion of eNOS increases L/E- interactions in the microvasculature [15], [16]. Further, NO decreases the expression of endothelial surface adhesion molecules, including P-selectin and VCAM-1 [17], [18]. In apoE^−/−^ atherosclerosis, L/E-interactions are increased, compared to C57BL/6J controls [19]. However, whether eNOS additionally modulates L/E-interactions in atherosclerosis has not been directly assessed.

In addition, NO is a potential regulator of platelet activation, platelet aggregation and P/E-interactions and multiple lines of evidence suggest that platelets promote atherosclerosis [20], [21], [22]. In hypercholesterolemic animal models, platelet adhesion to the endothelium occurs before plaques are visible [19], [23]. Platelets adhere to endothelial von Willebrand Factor (vWF) via glycoproteins (GP) and blockade of platelet GPIbα profoundly reduces lesion formation in apoE^−/−^ mice [19]. Whether eNOS influences P/E-interactions in atherosclerotic vessels on top of the changes reported in apoE^−/−^ compared to C57BL/6J wild type mice is currently unknown.

Because of the changes in gene expression and the alternative chemistry of NOS in atherosclerosis (NO vs. superoxide production), the role of each NOS isoform on key events of atherosclerosis can not be predicted. The anti-atherosclerotic and anti-adhesive properties of NO depend on its bioavailability, which is determined by the balance between its synthesis and degradation. In situations of increased oxidative stress, low NO bioavailability can result from NO's reaction with superoxide, giving rise to peroxynitrite formation [24], which in turn contributes to oxidative stress [25]. Oxidation of the NOS cofactor tetrahydrobiopterin (BH_4_) causes “uncoupling” of eNOS in vitro and may be a major source of vascular superoxide production promoting atherosclerosis [26], [27], [28].

The current study investigates the specific contribution of eNOS to L/E- and P/E-interactions in atherosclerosis. The effects of eNOS deletion on total vascular NO and superoxide productions in atherosclerotic apoE^−/−^ mice were studied with an aim to determine which of these two radicals largely determine the key events in atherosclerosis. Our results show that eNOS plays a major role in protection against atherosclerosis despite the fact that the enzyme is “uncoupled” and contributes to relevant superoxide as well as nitric oxide production.

## Methods

### Ethics statement

All procedures performed were approved by the ethics committee of University of Würzburg (Approval No. 54-2531.01-40/07).

### Mice

Animals were backcrossed for 10 generations to the C57BL/6J genetic background. eNOS^−/−^, provided by Paul Huang [10], and apoE^−/−^ (Jackson Laboratories, Bar Harbor, ME, USA) were crossed to generate double heterozygous mice. Offsprings were crossed and progenies were genotyped for eNOS by southern blotting and for apoE by polymerase chain reaction. apoE^−/−^ and apoE^−/−^/eNOS^−/−^ were weaned at 21 days and fed a western-type diet for 18 weeks (42% of total calories from fat; 0.15% cholesterol; Harlan Teklad, USA). For clear visualisation of L/E-interactions through the vessel wall, IVM studies were performed after 10 weeks of western-type diet, prior to macroscopic lesion development in this vessel segment. Animals were maintained in pathogen free facility with 12 hours light/dark cycle.

### Assessment of L/E and P/E- interactions by IVM studies

L/E- and P/E-interactions were assessed *in vivo* by use of video fluorescence microscopy [19]. Mice were anesthetized by intraperitoneal injection of a solution of midazolame (5 mg/kg body weight; Ratiopharm), medetomidine (0.5 mg/kg body weight; Pfizer), and fentanyl (0.05 mg/kg body weight; CuraMed Pharma GmbH). Due to easy accessibility, the carotid bifurcation was chosen for IVM studies. The right common carotid artery was carefully exposed, from 3 mm distal to 7 mm proximal to the carotid bifurcation. Tissues were continuously superfused with a thermostated bicarbonate-buffered saline solution, equilibrated with 5% CO_2_ in nitrogen, to maintain a physiological pH. Polyethylene catheters were implanted into the right jugular vein for intravenous injections.

For platelet isolation, blood was collected from the retro-orbital venous plexus of a donor mouse using heparin containing syringes. Blood was centrifuged at 400 g for 5 minutes. The supernatant was centrifuged at 250 g for 6 minutes to obtain platelet rich plasma. Subsequently, the platelets were isolated by centrifugation of the platelet rich plasma. The platelet pellet was re-suspended in PBS (pH 7.4) and incubated for 2 minutes with the fluorochrome carboxyfluorescein diacetate succinimidyl ester (CFSE, Molecular Probes). Labelled platelets were centrifuged and pellets were re-suspended in PBS and stored at room temperature until use. The suspension was adjusted to a final concentration of 50×10^6^ platelets per 250 µl and infused intravenously into a recipient mouse. As reported earlier, platelet preparation did not increase P-selectin expression, indicating absence of platelet activation due to preparation procedures [29]. Leukocytes were stained *in vivo* by intravenous injection of 100 µl 0.02% rhodamine 6G (Molecular Probes). The carotid artery was visualized using a Zeiss Axiotech microscope (water immersion objective: 20×, W 20×/0.5; Zeiss) with a mercury lamp for epi-illumination. The experiments were done as previously described [19]. L/E- and P/E-interactions were determined at high magnification (500-fold) in a 200 µm×100 µm area. L/E- and P/E-interactions were studied 200 µm proximal to the carotid bifurcation, at a predilection site for plaque development, which did not show macroscopic lesions at the time of IVM.

All images were videotaped and evaluated off-line, using a computer-assisted image analysis program (Cap Image 7.1; Dr. H. Zeintl, Ingenieurbüro Dr. Zeintl, Heidelberg, Germany). The cells that make an initial contact with the vessel wall followed by slow surface translocation (with several further contacts) with a velocity significantly lower than the centreline velocity were defined as ‘rolling cells’. Transiently adherent cells were defined as cells crossing an imaginary perpendicular through the vessel at a velocity significantly lower than the centreline velocity and were quantified as cells per mm^2^ endothelial surface. Cells that did not move or detach from the endothelial surface within 20 seconds of observation were defined as firmly adherent cells. All experiments were performed by a blinded operator.

### Evaluation of VCAM-1 RNA expression

Total RNA was isolated from the carotid arteries using a RNA Miniprep Kit (Stratagene, CA, USA). cDNA was synthesised from total RNA using the First Strand cDNA synthesis kit (Fermentas GmbH, Germany). mRNA expression of VCAM-1 and CD14 was quantified by real-time PCR (iCycler, Bio-Rad Laboratories, USA). PCR amplification was performed for 40 cycles at primer annealing of 60°C. VCAM-1 and CD14 expression was normalized to the HPRT signal. VCAM-1 sense: 5′-CTT GTG TTG AGC TCT GTG GGT TT-3′ and antisense: 5′-CAA TCT CCA GAT GGT CAA AGG GAT A -3′. CD14 sense: 5′- TAC CGA CCA TGG AGC GTG TG-3′ and antisense: 5′-GCC GGT TAC CTC GAG ATT TT-3′. HPRT sense: 5′- GTT GGA TAC AGG CCA GAC TTT GT-3′ and antisense: 5′- CCA CAG GAC TAG AAC ACC TGC-3′. Fluorogenic probes for VCAM-1: 5′-6FAM- CTG TGC AGT TGA CAG TGA CAG GTC TCC C XT –PH; CD14: 5′-6FAM –TGT TGC TTC TGG TGC ACG CCT CT –TMR; HPRT: 5′-6FAM-CTC GTA TTT GCA GAT TCA ACT TGC GC XT –PH were used. All primers and probes were obtained from TIB Molbiol, Germany.

### Histochemistry and immunohistochemistry

The area of the carotid arteries studied by IVM was stained with Oil red O. Additionally, immunohistochemistry for VCAM-1 and macrophages were done. The carotid arteries isolated from the anesthetized animals were embedded in Tissue-Tek® (Sakura Finetek, NL) and snap-frozen in liquid nitrogen. 5 µm sections were cut and air dried. Sections were stained with Oil red O or fixed in acetone for immunohistochemistry. Immunostaining for macrophage was performed using a mouse macrophage/monocyte monoclonal primary antibody (MOMA-2, Chemicon Int.) and for VCAM-1 using an anti-mouse VCAM-1 antibody (R&D systems). The macrophage and VCAM-1 stainings were visualised using DAB (Vector laboratories).

### Histomorphometry

Photomicrographs of the carotid artery were taken with a Leitz-camera mounted on a light microscope (Carl-Zeiss, Jena, Germany). Pictures were digitalized and transferred to a PC for planimetry using Image Pro Plus (Version 4.1; Media Cybernetics). All images were analysed at 400-fold magnification. Macrophage positive areas and lipid rich areas in the common carotid artery, in an area corresponding to the one used for IVM studies, were measured. Results were expressed as % positively stained plaque area.

### Double immunofluorescence

To investigate the cellular compartment of VCAM-1 expression, cryosections of the aortic arch of apoE^−/−^ and apoE^−/−^/eNOS^−/−^ mice were fixed with acetone. Following blocking procedures, cross reactivity of secondary antibodies with the alternating primary antibodies was ruled out. Expression of VCAM-1 protein in endothelial and smooth muscle cells was examined by double immunofluorescence. Slides were incubated with rat anti-VCAM-1 primary (BD Bioscience, 1∶20) followed by biotinylated rabbit anti-rat secondary antibody (Vector, 1∶50) and subsequently stained with streptavidin-texas red complex. After washing, slides were incubated with the antibodies directed against endothelial cells (rat anti-mouse CD 31, BD Bioscience, 1∶100) or vascular smooth muscle cells (α-actin, Sigma, 1∶60). The latter antibodies were directly labelled with fluoresceinisothiocyanate (FITC, green). Finally, all sections were mounted with 4′,6-diamidino-2-phenylindole (DAPI) mounting media and examined with a confocal microscope (Zeiss).

### Hemodynamics of the carotid circulation

The animals were anesthetized and the common carotid artery was visualized by duplex colour ultrasonography using an ultrasound system with a 15 MHz transducer (NICE, Toshiba Medical Systems, The Netherlands). The resistance index of the common carotid artery was calculated as the difference between the maximum systolic (V_sys_) and the end-diastolic flow velocity (V_dia_) divided by V_sys_.

### Measurement of vascular NO production by electron paramagnetic spintrapping

Detection of NO in the aorta was performed using colloid iron (II) diethyldithiocarbamate (Fe(DETC)_2_) according to a method which we adapted for detection of baseline NO production in aortic rings of apoE^−/−^ mice [30]. Briefly, animals were anesthetized with pentobarbital (80 µg/kg i.p.) and the aorta was perfused with 2 ml of Krebs-Hepes Buffer (KHB) through the left ventricle. The aorta was removed rapidly and placed in a petridish containing KHB. Perivascular fatty tissue was removed while the aorta was maintained at 4°C in KHB using a cold plate (Noxygen Science Transfer & Diagnostics, Denzlingen, Germany). The aorta was cut into 2 mm rings and placed in one well of a 24 well plate containing KHB. Subsequently, the aortic rings were incubated in the colloidal Fe-(DETC)_2_ spin trap solution for 1 hour. The spin trap was freshly prepared by mixing equal amounts of deoxygenated 1.6 mM FeSO_4_ and 3.2 mM DETC solutions. For pharmacological NOS inhibition samples were incubated with the NOS inhibitor, NG-nitro-L-arginine methyl ester (L-NAME) for 30 minutes at 37°C prior to the addition of spin trap. ESR measurements were done using a bench top e-scan ESR spectroscope (Bruker BioSpin GmbH, Germany). The instrumental settings were as follows: Centre field: 3308 G. Sweep width: 80 G. Microwave frequency: 9.495 GHz. Microwave power: 50 mW. Modulation Amplitude: 4.6 G. Modulation frequency: 86 kHz. Time constant: 81.92 ms. Conversion Time: 20.48 ms. Number of scans: 100. The protein content of the samples was quantified using a BCA protein assay kit (Pierce, IL, USA).

### Measurement of NO bioavailability in the blood stream

Nitrosyl hemoglobin, a reaction product of deoxygenated hemoglobin with NO is an in vivo marker for NO bioavailability in the circulation [31] and can be detected as a characteristic triplet peak by ESR spectroscopy. Blood samples were prepared according to a published method [32]. Briefly, the venous blood was drawn from the right ventricle. Following centrifugation at 2000 g (Eppendorf AG, Germany) the red cell cast was frozen in syringes and transferred into a finger dewar containing liquid nitrogen. The spectra were acquired using an X-band EMX spectroscope (Bruker BioSpin GmbH, Germany) with the following instrument settings: Centre field: 3340 G. Sweep width: 230 G. Microwave frequency: 9.452 GHz. Microwave power: 47.6 mW. Modulation Amplitude: 4.76 G. Modulation frequency: 86 kHz. Time constant: 40.96 ms. Conversion Time: 10.24 ms. Number of scans: 24. The NO concentration was determined from a calibration curve generated by incubating blood samples with known concentrations of nitrite and sodium dithionite (Na_2_S_2_O_4_).

### Measurement of intracellular superoxide production by HPLC-detection of oxyethidium

Superoxide production was measured by incubating vessel rings in KHB containing 50 µM dihydroethidium for 1 hour at 37°C [33]. Aortic rings were then homogenized in ice cold methanol, filtered and separated by reverse phase High Performance Liquid Chromatography (HPLC) using a C-18 column (Nucleosil 250, 4.5 mm; Sigma-Aldrich) along with an AKTA HPLC system (Amersham Biosciences, GE Healthcare). Oxyethidium, the reaction product of superoxide and dihydroethidium was quantitated with a fluorescence detector (Jasco, UK) at an excitation wavelength of 510 nm and emission wavelength of 595 nm. The amount of oxyethidium formed was normalised to the protein content of the samples.

### Measurement of vascular superoxide production by ESR

Production of superoxide was measured in aortic rings according to a previously published protocol [34] using the above mentioned e-scan spectroscope. Superoxide production was assessed by pre incubating aortic rings with PEG-SOD (100 U/ml) parallel to the spin trap 1-hydroxy-3-methoxycarbonyl-2,2,5,5-tetramethylpyrrolidine (CMH) in KHB for 1 hour at 37°C. The conversion of CMH to CM. radical in the PEG-SOD untreated samples was used to determine the total production of reactive oxygen species (ROS). The instrumental settings were as follows: Centre field: 3388 G. Sweep width: 132 G. Microwave frequency: 9.497 GHz. Microwave power: 1.25 mW. Modulation Amplitude: 1.63 G. Modulation frequency: 86 kHz. Time constant: 40.96 ms. Conversion Time: 10.24 ms. Number of scans: 50. The intensity of the ESR signal was normalized to the sample's protein content using a BCA protein assay kit.

### Western blot analysis

Aortic protein was isolated using RIPA buffer and western blots were performed using a monoclonal rabbit anti-eNOS, anti-nNOS (BD Biosciences) and a polyclonal anti-iNOS (Santa Cruz Biotechnology, Inc.) antibodies. To confirm equal loading of protein a goat anti-α-actin-antibody (Santa Cruz Biotechnology, Inc.) was used. Blots were analysed densitometrically using Scan Pack software (Biometra, Gottingen, Germany) and the density of protein bands for iNOS and nNOS were normalised to the corresponding α-actin bands of the same blots. Low temperature SDS-PAGE followed by western blot was done as mentioned previously for eNOS dimer/monomer protein detection [35].

### Statistical analyses

All data were expressed as mean±SE. Two way ANOVA was used for repeated measures, followed by Scheffe's F-test (Stat View 4.51, Abacus Concepts, Inc., Berkley, CA, USA). Student's *t*-test was used for unpaired data. A probability value of p≤0.05 was considered significant.

## Results

### Genetic deletion of eNOS increases L/E-interactions in apoE ^−/−^ mice

**Figure 1:**
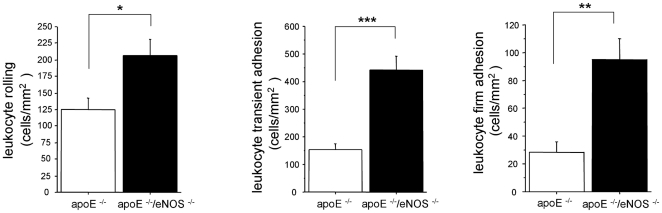
L/E-interactions analysed by intravital microscopy. The number of rolling, transiently adherent and firmly adherent leukocytes was significantly increased in the common carotid artery of apoE^−/−^/eNOS^−/−^ (n = 16), vs. apoE^−/−^ controls (n = 23), a) *p<0.01; b) ***p<0.0001; c) **p<0.001).

We used IVM to test whether eNOS derived NO affects L/E-interactions and whether these changes occur prior to macroscopic lesion formation. L/E-interactions were monitored in the common carotid artery, at a predilection site for plaque development that did not show macroscopic atherosclerotic lesions at the time of study. The number of rolling leukocytes was significantly higher in apoE^−/−^/eNOS^−/−^ (206±24 cells/mm2, n = 16), compared to apoE^−/−^ (125±17 cells/mm2, n = 23, p<0.01, Figure 1a). The number of transiently adherent leukocytes was higher in apoE^−/−^/eNOS^−/−^ (442±50 cells/mm2, n = 16), compared to apoE−/− (155±18 cells/mm2, n = 23, p<0.0001, Figure 1b). Finally, leukocyte firm adhesion was highly elevated in apoE^−/−^/eNOS^−/−^ (94±15 cells/mm2, n = 16), compared to apoE^−/−^ controls (28±8 cells/mm2, n = 23, p = 0.0002, Figure 1c).

### eNOS deficiency does not modulate platelet adhesion in apoE ^−/−^ mice

We investigated P/E-interactions to determine if eNOS influences platelet activation and adherence in a hyperlipidemia driven model of spontaneous atherosclerosis. Our results show no significant difference in the number of rolling platelets between apoE^−/−^ (53±12 cells/mm2, n = 19) and apoE^−/−^/eNOS^−/−^ (41±10 cells/mm2, n = 16, p = 0.48). The amount of transiently adherent platelets (apoE^−/−^: 505±57 cells/mm2, n = 9 vs. apoE^−/−^/eNOS^−/−^: 417±65 cells/mm2, n = 9, p = 0.33) and firmly adherent platelets (apoE^−/−^: 42±10 cells/mm2, n = 19 vs. apoE^−/−^/eNOS^−/−^: 20±13 cells/mm2, n = 16, p = 0.2, figures not shown) were similar.

### eNOS deficiency increases the expression of VCAM-1

**Figure 2:**
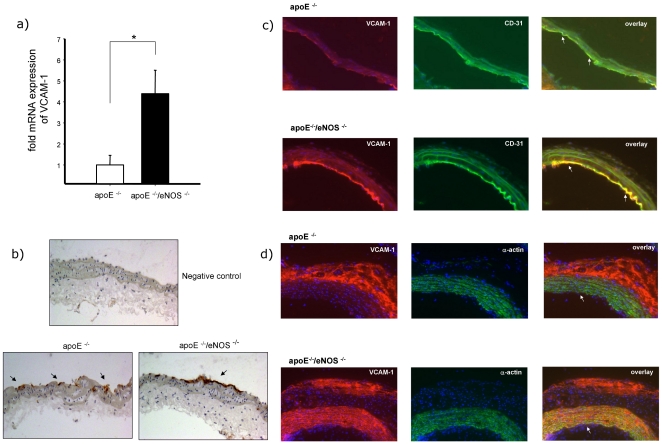
eNOS deletion increases VCAM-1 expression. a) Real time PCR analysis showed four fold increased expression of VCAM-1 mRNA in apoE^−/−^/eNOS^−/−^ (n = 9) carotids, compared to apoE^−/−^ (n = 20, *p<0.01). b) Immunohistochemistry confirmed increased endothelial VCAM-1 expression in carotid arteries of apoE^−/−^/eNOS^−/−^, compared to apoE^−/−^. Arrows indicate positive DAB staining (internal carotid artery, location of IVM). c) Double immunofluorescence staining of VCAM-1 protein in atherosclerotic lesions. Sections of the aortic arch of apoE^−/−^ and apoE^−/−^/eNOS^−/−^ animals were incubated with anti-VCAM-1 antibody (red) and anti-CD31 antibody (endothelial cells, green). Arrows indicate localization of VCAM-1 in endothelial cells in the overlay (yellow). Increased endothelial expression of VCAM-1 was observed in apoE^−/−^/eNOS^−/−^ compared to apoE^−/−^. d) Increased medial smooth muscle cell expression of VCAM-1 was observed in advanced plaques in the aortic arch in apoE^−/−^/eNOS^−/−^ compared to apoE^−/−^, as shown in yellow (arrows) by the double immunofluorescence staining of VCAM-1 (red) and smooth muscle cells (green).

In order to determine if eNOS deletion increases VCAM-1 expression we performed real time PCR experiments using total RNA samples of carotid arteries. Our results revealed that the mRNA expression of VCAM-1 was significantly higher in apoE^−/−^/eNOS^−/−^ animals, compared to apoE^−/−^ controls (4.3±1.1, n = 9 vs. 1.0±0.45, n = 20, p<0.002, Figure 2a). Additionally, immunohistochemistry showed increased endothelial VCAM-1 expression in apoE^−/−^/eNOS^−/−^ animals vs. apoE^−/−^ (Figure 2b). In order to check the cellular compartment of VCAM-1 expression, we performed double immunofluorescence in the aortic arch. We observed increased expression of VCAM-1 both in the endothelial (CD31 positive cells, Figure 2c) and medial smooth muscle cells (α-actin positive cells, Figure 2d) of apoE^−/−^/eNOS^−/−^ compared to apoE^−/−^ samples.

### NO from eNOS influences macrophage infiltration into the vessel wall

**Figure 3:**
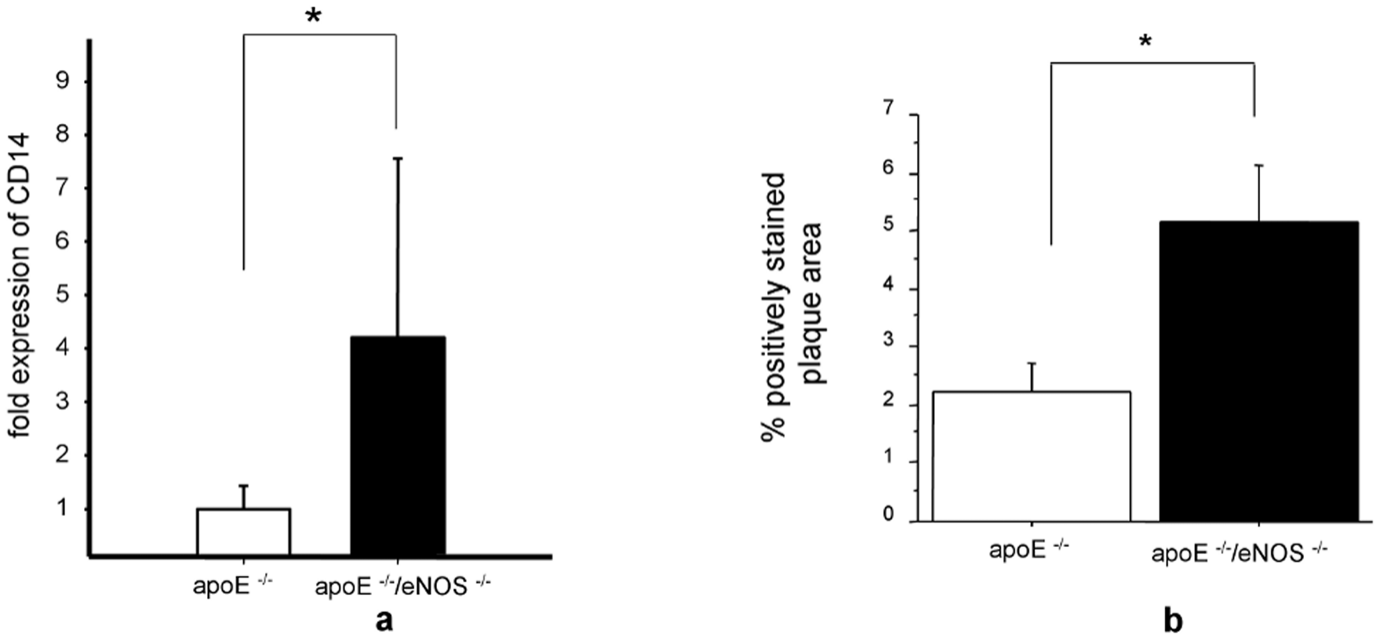
NO from eNOS influences macrophage infiltration in the vascular wall. a) Real time PCR analysis of CD14 showed significantly increased expression of CD14 mRNA in apoE^−/−^/eNOS^−/−^ (n = 12) carotids, compared to apoE^−/−^ (n = 17, *p<0.00002). b) Immunohistochemistry for MOMA-2 showed elevated vascular macrophage infiltration in carotid arteries of apoE^−/−^/eNOS^−/−^ (n = 10), compared to apoE^−/−^ (n = 9, *p<0.05).

To evaluate whether increased VCAM-1 expression in the carotid artery results in increased mononuclear leukocyte infiltration into the vessel wall we assessed CD14 expression by real time PCR. apoE^−/−^/eNOS^−/−^ showed a striking increase in CD14 expression, compared to apoE^−/−^ controls (4.2±3.3, n = 12 vs. 1.0±0.43, n = 17, p<0.00002, Figure 3a). This finding was supported by an increase of the macrophage positive staining areas in vessels of apoE^−/−^/eNOS^−/−^ (5.1±0.98% positively stained plaque area, n = 10), compared to apoE^−/−^ (2.2±0.48% positively stained plaque area, n = 9, p = 0.02, Figure 3b). Planimetry of the Oil red O positive, lipid rich areas in the vessel wall proximal to the carotid bifurcation showed no significant difference between apoE^−/−^ (8.1±4.0% positively stained plaque area, n = 9) and apoE^−/−^/eNOS^−/−^ animals (5.0±2.5% positively stained plaque area, n = 7, p = 0.56). The latter finding documents similar amounts of fatty streaks in the area of carotid artery chosen for IVM measurements.

### Unaltered vascular resistance index in eNOS deficiency

**Figure 4:**
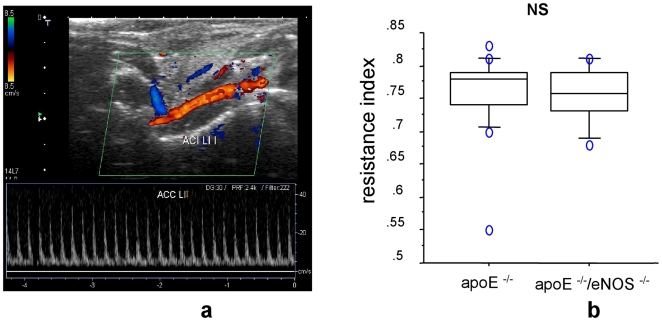
Unaltered vascular resistance index in eNOS deficiency. a) Representative picture of duplex ultrasonography in carotid arteries. b) Equal resistance index of carotid arteries from apoE^−/−^, n = 17, vs. apoE^−/−^/eNOS^−/−^, n = 10, p = 0.88, by duplex ultrasonography. NS denotes non-significance.

Duplex ultrasonography of the common carotid artery revealed that the resistance index in both, the apoE^−/−^ (0.758±0.015, n = 17) and apoE^−/−^/eNOS^−/−^ (0.754±0.014, n = 10, p = 0.88, Figure 4a and b) did not differ. These results show that at the time of the study, genetic deletion of eNOS did not change the hemodynamics of the carotid circulation.

### eNOS is a significant source of vascular wall NO production and circulating NO

**Figure 5:**
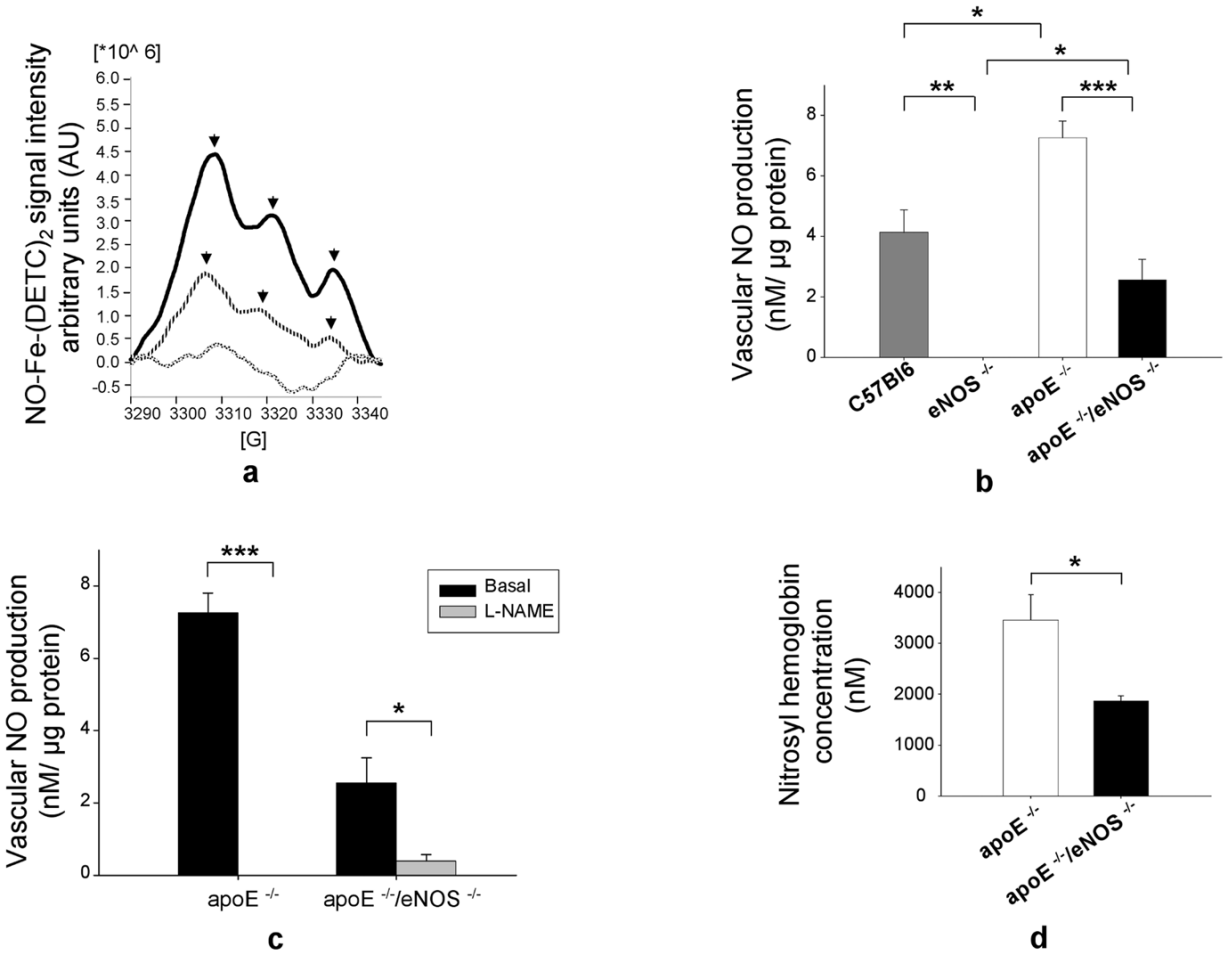
eNOS is a significant source of vascular wall NO production and circulating NO. a) ESR spectrum of NO-Fe-(DETC)_2_ in aortas of apoE^−/−^ and apoE^−/−^/eNOS^−/−^. Bold lines indicate apoE^−/−^, stripped lines apoE^−/−^/eNOS^−/−^ and patterned lines buffer/spin trap alone. Arrows show the typical 3 peaks NO-Fe-(DETC)_2_ signal. b) Vascular NO production in C57BL/6J (n = 12), eNOS^−/−^ (n = 8), apoE^−/−^ (n = 14) and apoE^−/−^/eNOS^−/−^ (n = 15), *p≤0.01, **p<0.001, ***p<0.0001). c) Vascular NO production with NOS inhibition using L-NAME in apoE^−/−^ (n = 11) and apoE^−/−^/eNOS^−/−^ mice (n = 16), *p<0.01, ***p<0.0001. d) Nitrosyl hemoglobin concentration of blood samples from apoE^−/−^/eNOS^−/−^ (n = 11) vs. apoE^−/−^ controls (n = 13, *p = 0.01).

Quantitation of baseline NO production in the vasculature is a challenging task, due to the radical's short half-life and very low bio available concentrations of NO. We used ESR, a method of highest sensitivity and specificity, in order to measure vascular NO production and NO levels in blood. Spin trapping of NO with colloidal Fe-(DETC)_2_ was used to measure baseline NO production of the vessel wall [30]. In addition, the paramagnetic properties of nitrosyl hemoglobin were utilised along with the ESR technique to quantify the *in vivo* concentrations of NO in whole blood [31]. Baseline vascular NO production was significantly lower in apoE^−/−^/eNOS^−/−^ (2.6±0.7 nM/µg protein, n = 15) than in apoE^−/−^ (7.3±0.6 nM/µg protein, n = 14, p<0.0001, Figure 5a and b). NO levels were significantly higher in apoE^−/−^ (7.3±0.6 nM/µg protein, n = 14) compared to C57BL/6J vessels (4.1±0.7 nM/µg protein, n = 12, p<0.01, Figure 5b). As expected, no ESR signal was detected in conventional eNOS^−/−^ mice. Interestingly, baseline NO production was significantly higher in apoE^−/−^/eNOS^−/−^(2.6±0.7 nM/µg protein, n = 15) compared to eNOS^−/−^ (0.0±0 nM/µg protein, n = 8, p≤0.01, Figure 5b) vessels. Inhibition of NOS using NG-nitro-L-arginine methyl ester (L-NAME) resulted in a significant reduction of vascular NO levels both in apoE^−/−^ (0.0±0 nM/µg protein, n = 11, vs. basal, p<0.0001, Figure 5c) and apoE^−/−^/eNOS^−/−^ mice (0.4±0.2 nM/µg protein, n = 17 vs. basal, p<0.01, Figure 5c). Additionally, nitrosyl hemoglobin levels in blood samples, reflecting bioavailable NO in the circulation, were reduced in apoE^−/−^/eNOS^−/−^ (1868±100 nM, n = 11), compared to apoE^−/−^ controls (3463±491 nM, n = 13, p<0.01, Figure 5d).

### eNOS is uncoupled and contributes to vascular production of superoxide in apoE^−/−^ mice

**Figure 6:**
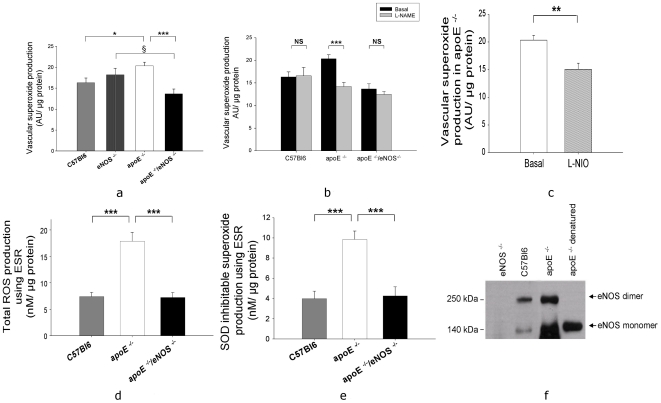
eNOS is uncoupled and contributes to vascular production of superoxide in apoE^−/−^ mice. a) HPLC measurements showed lower levels of superoxide production in apoE^−/−^/eNOS^−/−^ (n = 13) vs. apoE^−/−^ (n = 23). Superoxide levels were higher in apoE^−/−^ (n = 23) compared to C57BL/6J (n = 14). Interestingly, superoxide levels were significantly lower in apoE^−/−^/eNOS^−/−^ (n = 13) compared to eNOS^−/−^ (n = 12). b) L-NAME inhibited superoxide production in apoE^−/−^ (n = 15) but not in C57BL/6J (n = 17) and apoE^−/−^/eNOS^−/−^ (n = 12) aortas. c) Specific inhibition of eNOS using L-NIO resulted in significant reduction of superoxide production in apoE^−/−^ (n = 19). d) Total ROS production using ESR showed a significant increase in ROS levels in apoE^−/−^ (n = 23) compared to C57BL/6J (n = 12) and apoE^−/−^/eNOS^−/−^ (n = 15). e) Consistently, SOD inhibitable superoxide production measured by ESR also showed significant increase in superoxide levels in apoE^−/−^ (n = 23) compared to C57BL/6J (n = 12) and apoE^−/−^/eNOS^−/−^ (n = 15). ^§^p<0.05, *p<0.01, **p<0.001, ***p<0.0001, NS denotes non-significance. f) Uncoupling of eNOS in apoE^−/−^ compared to C57BL/6J aorta shown by western blot of eNOS protein dimer/monomer.

The HPLC measurements revealed higher vascular superoxide levels in apoE^−/−^ compared to apoE^−/−^/eNOS^−/−^ (20.3±0.9 AU/µg protein, n = 23, vs. 13.6±1.1 AU/µg protein, n = 13, p<0.0001, Figure 6a). Total superoxide formation in apoE^−/−^ was significantly increased compared to C57BL/6J (20.3±0.9 AU/µg protein, n = 23 vs. 16.3±1.1 AU/µg protein, n = 14, p<0.01, Figure 6a). Superoxide levels were not significantly different between C57BL/6J and eNOS^−/−^ mice (16.3±1.1 AU/µg protein, n = 14 vs. 18.2±1.5 AU/µg protein, n = 12, p>0.05, Figure 6a). Interestingly, genetic deletion of eNOS in apoE^−/−^ resulted in a significant reduction of superoxide levels compared to genetic deletion of eNOS in C57BL/6J mice (apoE^−/−^/eNOS^−/−^ vs. eNOS^−/−^: 13.6±1.1 AU/µg protein, n = 13 vs. 18.2±1.5 AU/µg protein, n = 12, p<0.05, Figure 6a), suggesting that eNOS contributes to superoxide production only in atherogenic mice. In addition to these results from our genetic model of chronic eNOS deficiency, acute pharmacological inhibition of NOS by L-NAME resulted in a significant reduction of superoxide in apoE^−/−^ (14.2±0.9 AU/µg protein vs. basal, n = 15, p<0.0001, Figure 6b) but not in C57BL/6J (16.6±1.8 AU/µg protein, n = 17 vs. basal, p>0.05) and apoE^−/−^/eNOS^−/−^ mice (12.4±0.6 AU/µg protein, n = 12 vs. basal, p>0.05), suggesting that NOS uncoupling contributes to oxidative stress in apoE^−/−^. Moreover, L-NIO, a specific eNOS inhibitor significantly decreased superoxide production in apoE^−/−^, supporting partial uncoupling of eNOS (15.0±1.1 AU/µg protein, n = 19 vs. 20.3±0.9 AU/µg protein, n = 23, p<0.001, Figure 6c). ESR measurements of total ROS production, measured by the conversion of CMH to CM. showed a significant increase in ROS levels in apoE^−/−^ (17.9±1.7 nM/µg protein, n = 23) compared to C57BL/6J (7.3±0.8 nM/µg protein, n = 12, p<0.0001) and apoE^−/−^/eNOS^−/−^ mice (7.2±0.9 nM/µg protein, n = 15, p<0.0001, Figure 6d). Consistently, measurements of SOD inhibitable superoxide levels using ESR showed significantly increased superoxide production in apoE^−/−^ (9.8±0.8 nM/µg protein, n = 23) compared to C57BL/6J (3.9±0.7 nM/µg protein, n = 12, p<0.0001, Figure 6e) and apoE^−/−^/eNOS^−/−^ mice (4.2±0.9 nM/µg protein, n = 15, p<0.0001, Figure 6e). The uncoupling of eNOS was confirmed by a western blot for eNOS dimer/monomer. The aortic lysates of C57BL/6J animals showed increased eNOS protein dimers while apoE^−/−^ animals showed high levels of eNOS protein monomers.

### Western blot analysis of NOS isoforms

**Figure 7:**
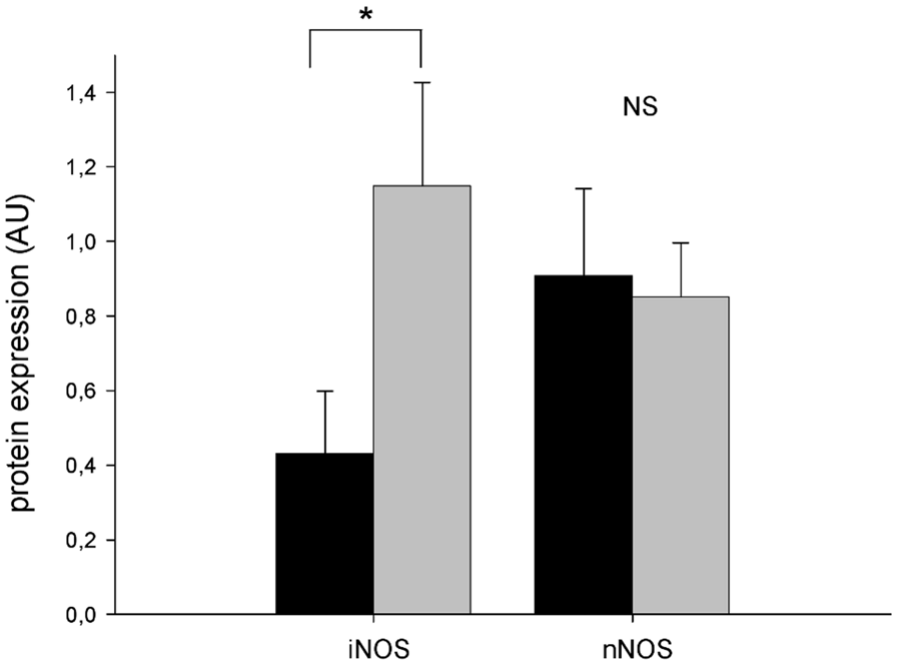
Vascular expression of NOS isoforms. Significantly increased expression of iNOS protein in the aorta of apoE^−/−^/eNOS^−/−^ (n = 10) compared to apoE^−/−^ mice (n = 10). The protein levels of nNOS did not differ between apoE^−/−^ (n = 10) apoE^−/−^/eNOS^−/−^ (n = 11). * p<0.05, NS denotes non-significance.

Genetic deletion of eNOS resulted in significant increase of iNOS protein expression in the aorta of apoE^−/−^ mice compared to controls (1.14+0.27, n = 10 vs. 0.43+0.16, n = 10, p<0.05, Figure 7). The expression levels of nNOS protein did not differ significantly between apoE^−/−^ and apoE^−/−^/eNOS^−/−^ mice (0.90+0.23, n = 10 vs. 0.85+0.14, n = 11, p>0.05, Figure 7).

## Discussion

Chronic eNOS deficiency significantly increases leukocyte rolling, transient and firm adhesion in apoE^−/−^/eNOS^−/−^, compared to apoE^−/−^ controls. Increased L/E-interactions were not due to differences in local plaque burden, as both genotypes showed similar amounts of fatty streaks in carotid artery segments used for IVM at an early time point. Duplex ultrasonography studies documented similar hemodynamics in the carotid circulation of both genotypes. Therefore, the increase in L/E-interactions in apoE^−/−^/eNOS^−/−^ animals is not secondary to alterations of flow dynamics.

To the best of our knowledge this is the first study investigating the role of eNOS in L/E-interactions in spontaneous hyperlipidemia driven atherosclerosis. We show that lack of endogenous NO production by eNOS accelerates L/E-interactions in apoE atherosclerosis. Our data is in line with previous studies showing that endogenous NO production inhibits L/E-interactions in the microvasculature [15], [16]. We previously reported that atherosclerosis is accelerated in apoE^−/−^/eNOS^−/−^ and that this increase in lesion formation is not secondary to increased blood pressure in this genotype [10], [36]. Therefore, increased atherosclerosis development in apoE^−/−^/eNOS^−/−^ animals is likely due to local changes like increased endothelial activation and L/E- interactions reported here.

VCAM-1 expression is a hallmark of endothelial cell activation [37]. In atherosclerosis, the interaction of macrophage very late antigen-4 (VLA-4) and endothelial VCAM-1 mediates monocyte firm adhesion. Upregulation of VCAM-1 expression following treatment of human saphenous vein endothelial cells with the NOS inhibitor L-NAME, suggests tonic suppression of adhesion molecule expression by NO [18]. In contrast, our study of microvascular endothelial cells from eNOS^−/−^ mice showed that eNOS deletion alone does not induce adhesion molecule expression or increase L/E-interactions, suggesting that eNOS serves a permissive role in endothelial activation [38]. The data presented here indicates that eNOS deletion increases VCAM-1 expression not only in the endothelium, causing endothelial activation and recruitment of leukocytes, but also in medial smooth muscle cells.

IVM studies in apoE^−/−^ mice suggest that P/E-interactions are an important trigger for plaque formation [19], [39], [40]. Since NO was shown to inhibit platelet activation and aggregation *in vitro*, we studied the importance of eNOS derived NO in the modulation of P/E-interactions in atherosclerosis. Our study shows no difference in platelet rolling, transient, or firm adhesion to the endothelium in apoE^−/−^/eNOS^−/−^ mice, compared to apoE^−/−^ controls. This suggests that the dramatically increased atherosclerosis in apoE^−/−^/eNOS^−/−^, compared to apoE^−/−^ [10], [41], is not caused by increased platelet adhesion and subsequent events. Therefore, although platelets accelerate plaque formation in apoE^−/−^ mice, they may not contribute to the increased plaque formation in apoE^−/−^/eNOS^−/−^.

Spin trapping of NO by Fe-(DETC)_2_ using ESR, considered the utmost specific and sensitive method for detection of NO in biological systems, showed that eNOS contributes significantly to total vascular NO production in atherosclerosis. We recently reported that the significant increase in vascular NO production observed in apoE^−/−^ compared to C57BL/6J mice is due to iNOS [42]. Interestingly, apoE^−/−^/eNOS^−/−^ mice had significantly higher NO levels compared to eNOS^−/−^ mice. This increase in NO production is likely mediated by iNOS since aortic iNOS protein expression was increased in apoE^−/−^/eNOS^−/−^ compared to apoE^−/−^ (Figure 7). In contrast, we did not observe changes in expression levels of nNOS in apoE^−/−^ and apoE^−/−^/eNOS^−/−^ (Figure 7). Consistent with the reduction of endothelial NO production, the *in vivo* concentration of circulating nitrosyl hemoglobin, reflecting bioavailable NO in the circulation, was markedly reduced in apoE^−/−^/eNOS^−/−^ mice. Considering the expression of all three NOS isoforms in atherosclerotic plaques, our results are not easily predictable because of several reasons. First, increased L/E-interactions, macrophage infiltration, increased vascular iNOS expression and plaque area secondary to eNOS deletion could have also resulted in increased cytotoxic NO formation by iNOS. Secondly, the deletion of eNOS could have changed the bioavailability of substrate and co-factor, thereby influencing the chemistry of iNOS and nNOS. Our data suggest that eNOS is an important source of vascular and circulating NO in apoE^−/−^ mice, despite all these possibilities. The residual NO detectable by the characteristic triplet ESR peak in apoE^−/−^/eNOS^−/−^ indicates residual NO production by iNOS and possibly nNOS in the vessel wall.

Our assessment of vascular superoxide formation using two independent techniques revealed a significant reduction of superoxide production following genetic eNOS deletion (chronic) in apoE^−/−^. Inhibition of NOS isoforms using L-NAME resulted in significant reduction of superoxide production in apoE^−/−^ but not in C57BL/6J and apoE^−/−^/eNOS^−/−^, suggesting that NOS is uncoupled only in atherogenic apoE^−/−^ mice. Additionally, acute pharmacological inhibition of eNOS using L-NIO reduced superoxide formation in apoE^−/−^. The reduction of vascular superoxide in apoE^−/−^/eNOS^−/−^ reflects the contribution of endothelial, eNOS derived superoxide production. Since both, significant NO and superoxide production by eNOS is detectable in apoE^−/−^ mice we conclude that eNOS is partially uncoupled during atherosclerosis development in this model. In support of this our eNOS western blots showed increased eNOS monomer, an indirect measure of eNOS uncoupling in apoE^−/−^ mice. As shown in eNOS over-expressing apoE^−/−^ mice, increased superoxide generation by uncoupled eNOS may accelerate atherosclerosis [43]. However, despite decreased vascular superoxide formation in our apoE^−/−^/eNOS^−/−^ model, these animals develop increased atherosclerosis, suggesting that the atheroprotective effect of decreased superoxide production does not outweigh the proatherogenic effects of NO deficiency. In other words, we made the intriguing observation that the increased eNOS derived superoxide formation does not override the damage caused by a concomitant decrease of NO production following eNOS deletion. Ample evidence implicates increased superoxide production in atherosclerosis [44], [45]. Our data is consistent with this hypothesis since we observed increased superoxide production in apoE^−/−^ compared to C57BL/6J animals. Therefore, eNOS uncoupling is a key source of superoxide production in the apoE^−/−^ model.

The major findings of our study indicate that lack of eNOS reduces vessel wall oxidative stress in atherosclerosis despite accelerated plaque formation, increased vascular inflammation and L/E-interactions in apoE^−/−^/eNOS^−/−^ vessels. P/E-interactions do not differ between apoE^−/−^ and apoE^−/−^/eNOS^−/−^, indicating that lack of NO production does not increase platelet interactions on top of the apoE^−/−^ background. Uncoupling of eNOS occurs in apoE^−/−^ atherosclerosis but does not negate the enzyme's strong protective effects. Hence, eNOS derived NO can still play a protective role in a system of high oxidative stress, potentially associated with increased peroxynitrite formation.

## References

[1] Lusis AJ (2000). Atherosclerosis.. Nature.

[2] Ross R (1993). The pathogenesis of atherosclerosis: a perspective for the 1990s.. Nature.

[3] Gawaz M, Langer H, May AE (2005). Platelets in inflammation and atherogenesis.. J Clin Invest.

[4] Davignon J, Ganz P (2004). Role of endothelial dysfunction in atherosclerosis.. Circulation.

[5] Wilcox JN, Subramanian RR, Sundell CL, Tracey WR, Pollock JS (1997). Expression of multiple isoforms of nitric oxide synthase in normal and atherosclerotic vessels.. Arterioscler Thromb Vasc Biol.

[6] Albrecht EW, Stegeman CA, Heeringa P, Henning RH, van Goor H (2003). Protective role of endothelial nitric oxide synthase.. J Pathol.

[7] Pou S, Pou WS, Bredt DS, Snyder SH, Rosen GM (1992). Generation of superoxide by purified brain nitric oxide synthase.. J Biol Chem.

[8] Xia Y, Roman LJ, Masters BS, Zweier JL (1998). Inducible nitric-oxide synthase generates superoxide from the reductase domain.. J Biol Chem.

[9] Xia Y, Tsai AL, Berka V, Zweier JL (1998). Superoxide generation from endothelial nitric-oxide synthase. A Ca2+/calmodulin-dependent and tetrahydrobiopterin regulatory process.. J Biol Chem.

[10] Kuhlencordt PJ, Gyurko R, Han F, Scherrer-Crosbie M, Aretz TH (2001). Accelerated atherosclerosis, aortic aneurysm formation, and ischemic heart disease in apolipoprotein E/endothelial nitric oxide synthase double-knockout mice.. Circulation.

[11] Kuhlencordt PJ, Hotten S, Schodel J, Rutzel S, Hu K (2006). Atheroprotective effects of neuronal nitric oxide synthase in apolipoprotein e knockout mice.. Arterioscler Thromb Vasc Biol.

[12] Kuhlencordt PJ, Chen J, Han F, Astern J, Huang PL (2001). Genetic deficiency of inducible nitric oxide synthase reduces atherosclerosis and lowers plasma lipid peroxides in apolipoprotein E-knockout mice.. Circulation.

[13] Detmers PA, Hernandez M, Mudgett J, Hassing H, Burton C (2000). Deficiency in inducible nitric oxide synthase results in reduced atherosclerosis in apolipoprotein E-deficient mice.. J Immunol.

[14] Kubes P, Suzuki M, Granger DN (1991). Nitric oxide: an endogenous modulator of leukocyte adhesion.. Proc Natl Acad Sci U S A.

[15] Sanz MJ, Hickey MJ, Johnston B, McCafferty DM, Raharjo E (2001). Neuronal nitric oxide synthase (NOS) regulates leukocyte-endothelial cell interactions in endothelial NOS deficient mice.. Br J Pharmacol.

[16] Lefer DJ, Jones SP, Girod WG, Baines A, Grisham MB (1999). Leukocyte-endothelial cell interactions in nitric oxide synthase-deficient mice.. Am J Physiol.

[17] Gauthier TW, Davenpeck KL, Lefer AM (1994). Nitric oxide attenuates leukocyte-endothelial interaction via P-selectin in splanchnic ischemia-reperfusion.. Am J Physiol.

[18] De Caterina R, Libby P, Peng HB, Thannickal VJ, Rajavashisth TB (1995). Nitric oxide decreases cytokine-induced endothelial activation. Nitric oxide selectively reduces endothelial expression of adhesion molecules and proinflammatory cytokines.. J Clin Invest.

[19] Massberg S, Brand K, Gruner S, Page S, Muller E (2002). A critical role of platelet adhesion in the initiation of atherosclerotic lesion formation.. J Exp Med.

[20] Michelson AD, Benoit SE, Furman MI, Breckwoldt WL, Rohrer MJ (1996). Effects of nitric oxide/EDRF on platelet surface glycoproteins.. Am J Physiol.

[21] Stamler J, Mendelsohn ME, Amarante P, Smick D, Andon N (1989). N-acetylcysteine potentiates platelet inhibition by endothelium-derived relaxing factor.. Circ Res.

[22] Cerwinka WH, Cooper D, Krieglstein CF, Feelisch M, Granger DN (2002). Nitric oxide modulates endotoxin-induced platelet-endothelial cell adhesion in intestinal venules.. Am J Physiol Heart Circ Physiol.

[23] Theilmeier G, Michiels C, Spaepen E, Vreys I, Collen D (2002). Endothelial von Willebrand factor recruits platelets to atherosclerosis-prone sites in response to hypercholesterolemia.. Blood.

[24] Huie RE, Padmaja S (1993). The reaction of no with superoxide.. Free Radic Res Commun.

[25] White CR, Brock TA, Chang LY, Crapo J, Briscoe P (1994). Superoxide and peroxynitrite in atherosclerosis.. Proc Natl Acad Sci U S A.

[26] Landmesser U, Dikalov S, Price SR, McCann L, Fukai T (2003). Oxidation of tetrahydrobiopterin leads to uncoupling of endothelial cell nitric oxide synthase in hypertension.. J Clin Invest.

[27] Vasquez-Vivar J, Kalyanaraman B, Martasek P, Hogg N, Masters BS (1998). Superoxide generation by endothelial nitric oxide synthase: the influence of cofactors.. Proc Natl Acad Sci U S A.

[28] Rabelink TJ, Luscher TF (2006). Endothelial nitric oxide synthase: host defense enzyme of the endothelium?. Arterioscler Thromb Vasc Biol.

[29] Massberg S, Eisenmenger S, Enders G, Krombach F, Messmer K (1998). Quantitative analysis of small intestinal microcirculation in the mouse.. Res Exp Med (Berl).

[30] Kleschyov AL, Mollnau H, Oelze M, Meinertz T, Huang Y (2000). Spin trapping of vascular nitric oxide using colloid Fe(II)-diethyldithiocarbamate.. Biochem Biophys Res Commun.

[31] Hall DM, Buettner GR (1996). In vivo spin trapping of nitric oxide by heme: electron paramagnetic resonance detection ex vivo.. Methods Enzymol.

[32] Dikalov S, Fink B (2005). ESR techniques for the detection of nitric oxide in vivo and in tissues.. Methods Enzymol.

[33] Fink B, Laude K, McCann L, Doughan A, Harrison DG (2004). Detection of intracellular superoxide formation in endothelial cells and intact tissues using dihydroethidium and an HPLC-based assay.. Am J Physiol Cell Physiol.

[34] Dikalova A, Clempus R, Lassegue B, Cheng G, McCoy J (2005). Nox1 overexpression potentiates angiotensin II-induced hypertension and vascular smooth muscle hypertrophy in transgenic mice.. Circulation.

[35] Schafer A, Fraccarollo D, Pfortsch S, Flierl U, Vogt C (2008). Improvement of vascular function by acute and chronic treatment with the PDE-5 inhibitor sildenafil in experimental diabetes mellitus.. Br J Pharmacol.

[36] Chen J, Kuhlencordt PJ, Astern J, Gyurko R, Huang PL (2001). Hypertension does not account for the accelerated atherosclerosis and development of aneurysms in male apolipoprotein e/endothelial nitric oxide synthase double knockout mice.. Circulation.

[37] Bevilacqua MP, Pober JS, Mendrick DL, Cotran RS, Gimbrone MA (1987). Identification of an inducible endothelial-leukocyte adhesion molecule.. Proc Natl Acad Sci U S A.

[38] Kuhlencordt PJ, Rosel E, Gerszten RE, Morales-Ruiz M, Dombkowski D (2004). Role of endothelial nitric oxide synthase in endothelial activation: insights from eNOS knockout endothelial cells.. Am J Physiol Cell Physiol.

[39] Massberg S, Schurzinger K, Lorenz M, Konrad I, Schulz C (2005). Platelet adhesion via glycoprotein IIb integrin is critical for atheroprogression and focal cerebral ischemia: an in vivo study in mice lacking glycoprotein IIb.. Circulation.

[40] Huo Y, Schober A, Forlow SB, Smith DF, Hyman MC (2003). Circulating activated platelets exacerbate atherosclerosis in mice deficient in apolipoprotein E.. Nat Med.

[41] Knowles JW, Reddick RL, Jennette JC, Shesely EG, Smithies O (2000). Enhanced atherosclerosis and kidney dysfunction in eNOS(−/−)Apoe(−/−) mice are ameliorated by enalapril treatment.. J Clin Invest.

[42] Ponnuswamy P, Ostermeier E, Schrottle A, Chen J, Huang PL (2009). Oxidative stress and compartment of gene expression determine proatherosclerotic effects of inducible nitric oxide synthase.. Am J Pathol.

[43] Ozaki M, Kawashima S, Yamashita T, Hirase T, Namiki M (2002). Overexpression of endothelial nitric oxide synthase accelerates atherosclerotic lesion formation in apoE-deficient mice.. J Clin Invest.

[44] Ohara Y, Peterson TE, Harrison DG (1993). Hypercholesterolemia increases endothelial superoxide anion production.. J Clin Invest.

[45] Sorescu D, Weiss D, Lassegue B, Clempus RE, Szocs K (2002). Superoxide production and expression of nox family proteins in human atherosclerosis.. Circulation.

